# Clinical experience with a new injectable hyaluronic acid designed to improve skin quality in a private clinic in Brazil: A retrospective cohort study

**DOI:** 10.1002/hsr2.399

**Published:** 2021-10-05

**Authors:** Daniel Dal'Asta Coimbra

**Affiliations:** ^1^ Instituto de Dermatologia Professor Rubem David Azulay Santa Casa de Misericórdia/IDPRDA Rio de Janeiro Brazil

**Keywords:** aesthetics, dermal fillers, hyaluronic acid, *Juvéderm* Volite, skin aging

## Abstract

**Background:**

Juvéderm Volite is a skin‐conditioning hyaluronic acid (HA) gel for intradermal injection that provides longer‐lasting effects with a lower concentration of hyaluronic acid. Few studies evaluating its use for aesthetic purposes are available.

**Aim:**

To examine the use and safety of Juvéderm Volite in daily clinical practice.

**Methods:**

A retrospective cohort study of subjects treated with Juvéderm Volite for aesthetic purposes from May 2018 to October 2019 in Rio de Janeiro. Data were extracted from the attending physician's records obtained at each medical appointment. Subjects were assessed according to their age group and treatment characteristics, which include the use of cannulas vs needles and the effectiveness and safety of associated treatments in a single session. Need for subsequent treatment was stratified by touch‐up treatment (<3‐month period) and repeat treatment (≥3‐month period). Safety assessment was based on the report of nodule formation and late hypersensitivity in patients. Appropriate statistical tests were used for data analysis.

**Results:**

One hundred and eight subjects were included in the study analysis. The total number of treatment sessions consisted of 159, with a mean follow‐up time of 300.3 days. Of the total 108 subjects, 8.4% required touch‐up treatment for optimum correction and repeat treatment occurred in 9.0%. No cases of adverse events were reported during the follow‐up period.

**Conclusions:**

This study has shown, based on clinical observation, that Juvéderm Volite is a useful tool to improve skin quality, requiring fewer and less frequent maintenance treatments. No serious adverse events were reported during the follow‐up period.

## 1 INTRODUCTION

Minimally invasive procedures using hyaluronic acid‐based dermal fillers have seen a significant rise over the years worldwide. According to the International Society of Aesthetic Plastic Surgery (ISAPS), hyaluronic acid injection is the second most frequently performed non‐surgical cosmetic procedure in the world, with over 3 million treatments carried out in the United States alone in 2017, approximately 15% higher than the number reported in 2015.^1^, ^2^ In this context, Brazil ranks second in terms of number of cosmetic procedures performed worldwide with more than 2 million treatments in 2017. If we consider only non‐surgical cosmetic procedures, hyaluronic acid injection was the second most common treatment in the country, with an estimated 254 375 procedures performed that same year.^1^

Despite existing evidence attesting to the efficacy and safety of hyaluronic acid‐based dermal fillers for aesthetic purposes and multiple HA products available for use in clinical practice, not much has been described regarding the clinical use of Juvéderm Volite in previous studies.

The prevention of complications associated with treatment depends on technical expertise and domain knowledge of the different products.^3^, ^4^, ^5^ Complications are generally rare and may be classified as early (<14 days), late (14 days to 1 year), and delayed (>1 year). Ecchymosis, edema, erythema, infection, allergic reaction, nodule formation, angioedema, skin necrosis, and embolism are examples of early‐onset complications; late complications include hyperpigmentation, infection, and granulomas; and *biofilm*‐associated infected nodules are a type of delayed‐onset complication.^6^ A study conducted in Brazil on the use of Juvéderm Volift for rhinomodulation showed that some patients experienced swelling and pain‐evoked touch (resolved 14 days after treatment) and mild adverse events such as hematoma and erythema.^7^

Currently available products differ in terms of hyaluronic acid concentration and technology. Juvéderm Volite is an injectable cross‐linked HA gel with lidocaine intended for intradermal injection and designed to improve skin quality attributes such as surface smoothness, hydration, and elasticity. The product uses Vycross technology (Allergan Inc.), which incorporates short‐chain HA together with long‐chain HA to provide more efficient crosslinking than fillers based on other technologies, with a lower concentration of hyaluronic acid and longer‐lasting results. The less hydrophilic gel makes it a safer product with more predictable and natural‐looking results.^7^ However, it is interesting to note that there are relatively few studies describing the efficacy and safety of Juvéderm Volite for aesthetic purposes, despite its widespread use in clinical practice.^8^, ^9^, ^10^

Ogilvie et al (2020) published an expert consensus on the use of Volite to treat fine lines recommending micro‐depot injections of Volite into the deep dermis with a 32G ½ needle inserted at <45° to the skin, spaced 0.5 to 1.0 cm apart, with 0.01 to 0.05 mL volume per injection (full‐face total volume: ~2 mL). Primary target areas of treatment were the malar, perioral, neck, and décolletage regions. The panel did not identify the forehead and dorsum of the hands as primary targets for Volite treatment. Small adjustments to the volume and spacing of injection may be recommended based on specific skin regions and patient characteristics.^11^

Studies published to date have all described the placement of Volite in the intradermal plane using a sharp needle.^8^, ^9^, ^10^ But would there be any difference in clinical outcomes and/or changes in injection technique if the filler were placed in the superficial subcutaneous tissue? To address the knowledge gap regarding the use of cannulas to inject Juvéderm Volite in the subcutaneous layer, other than injecting the product intradermally as recommended by existing clinical practical guidelines, the author conducted this retrospective study to investigate its use with both cannulas and needles in daily clinical practice, as well as to assess its safety and need for subsequent treatment.

## 2 MATERIALS AND METHODS

### 2.1 Study design

This is a retrospective cohort study of 108 patients treated with Juvéderm Volite for aesthetic purposes from May 2018 to October 2019 at the Les Peaux Dermatological Clinic, located in the city of Rio de Janeiro.

Data were extracted from the attending physician's records obtained at each medical appointment. Subjects were treated with Juvéderm Volite at the Les Peaux Dermatological Clinic according to individual needs and clinical indication. No pretreatment instructions were given to study participants. Consultations were documented in medical records during each visit, thus allowing retrospective data to be used by the investigator to track the clinical outcomes of patients during the follow‐up period.

The study was conducted in accordance with local applicable regulations and approved by the Research Ethics Committee (CAAE: 29001519.2.0000.5235). Given the retrospective nature of the study design, written informed consent was sought for all subjects.

### 2.2 Eligibility criteria

Convenience sampling was used as practical criteria for subject inclusion in the study. All patients who underwent treatment with Juvéderm Volite for aesthetic purposes from May 13, 2018 to October 11, 2019 at the Les Peaux Dermatological Clinic, located in the city of Rio de Janeiro, were included in the study sample. Individuals without clinical indication, with a known allergy to hyaluronic acid, pregnant or breastfeeding or having skin inflammatory conditions such as acne or erysipelas are not eligible for treatment with Juvéderm Volite and therefore were not considered for the study.

### 2.3 Data collection

Data were collected retrospectively by reviewing clinical records of patients treated with Juvéderm Volite for the duration of the study.

The following variables were collected from each treatment session: number of syringes, treatment area, needle or cannula use, size of cannula, combined treatments (ie, other hyaluronic acid dermal fillers, botulinum toxin, poly‐l‐lactic acid, calcium hydroxyapatite, superficial peeling, or laser), and reports of mild adverse events (*visible* or *palpable* mass) *or moderate adverse events* (nodule formation and late hypersensitivity).

### 2.4 Juvéderm Volite injection technique

**FIGURE 1:**
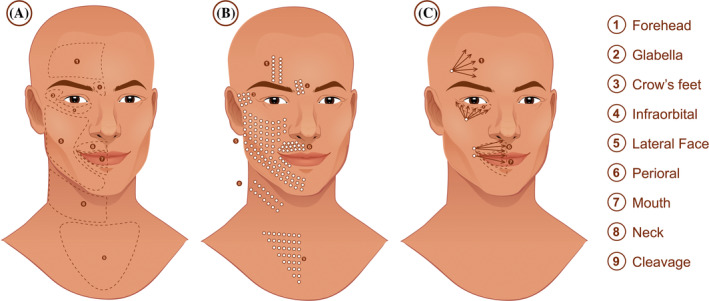
Treatment areas A; technique with a needle B; technique with a cannula C

Figure 1A shows treatment areas of injection with Juvéderm Volite recommended by the author. Injection technique with needle (Figure 1B); injection technique with cannula (Figure 1C).

Considering that Juvéderm Volite is intended for intradermal injections, the use of a needle is preferable. When using a cannula, injections should be administered in the superficial subcutaneous tissue, as close as possible to the dermis, regardless of the size of cannula. Tilting the cannula against the underside of the skin allows the injector to ascertain proper subdermal position.

Injection with a needle is performed with 0.01 cc microdroplets of product placed at each point. The distance between points varies according to the indication and area of treatment, as shown in Figure 1B. Most treatments are carried out with injections spaced 0.5 to 1.0 cm apart, except in procedures aimed at improving rhytids and linear depressions. In these cases, for example, space injections generally range from 0.2 to 0.3 cm apart in areas, such as glabella lines (area 2), vertical forehead lines (area 1), crow's feet (area 3), and horizontal neck lines (area 8). However, treatment of the neck must be restricted to horizontal lines, as treatment of the entire region may lead to undesired side effects such as lumps and bumps beneath the surface of the skin at the injection site.

Injection technique with a cannula can be seen in Figure 1C. Juvéderm Volite is deposited in a linear droplet fashion following the direction of the arrows, as close as possible to the dermis. The amount of product required when using a cannula is usually greater than when injecting with a needle. But, by contrast, the risk of bruising, especially in the perioral region (area 6), is greatly reduced, as well as other common complications like visible lumps and bumps that occur with filler placement in areas with scarce subcutaneous fat, such as the infraorbital region (area 4) and lateral forehead (area 1).

When treatment is specifically aimed at improving lip hydration or promoting a lip gloss‐effect (area 7), both needles and cannulas can be used. However, due to a lower risk of intravascular injection and bruising, cannulas are more commonly used.

### 2.5 Study endpoints

Besides descriptive analysis concerning the use of Juvéderm Volite for aesthetic purposes, two primary endpoints were defined: need for touch‐up treatment within 3 months of the initial treatment and repeat treatment 3 months after initial treatment. Need for subsequent treatment was defined by the attending physician based on subjective criteria, and therefore, it was solely up to the injector to decide whether the patient needed further treatment.

Safety was based on the reporting of treatment‐related adverse events (AEs), such as nodule formation and late hypersensitivity.

### 2.6 Statistical analysis

Frequency measures were used for categorical variables, and measures of central tendency and dispersion for numerical variables.

For demographic data, the analysis was performed considering the total number of patients in the sample and for treatment characteristics, the number of sessions was used as the unit of analysis. These were stratified by the sample size, age group (≤40 or >40 years old), and treatment with needle or cannula. Touch‐up treatment and repeat treatment variables were measured according to the number of sessions, gender, and age of patients.

A Mann‐Whitney *U* test (non‐normal distribution) and a *t* test (normal distribution) were used to assess the association between patient and treatment characteristics. The Shapiro‐Wilk test was used to check data normality. Correlation was used to verify the linear relationship between age and number of Volite syringes per area. In addition, a *χ* ^2^ was used to determine whether there is a pattern of dependence between patient and treatment characteristics (ie, treatment area, associated treatments, and injection technique—needle vs cannula). A 5% confidence level was considered. Software R version 3.6.1 and Microsoft Office 365 were used.

## 3 RESULTS

**FIGURE 2:**
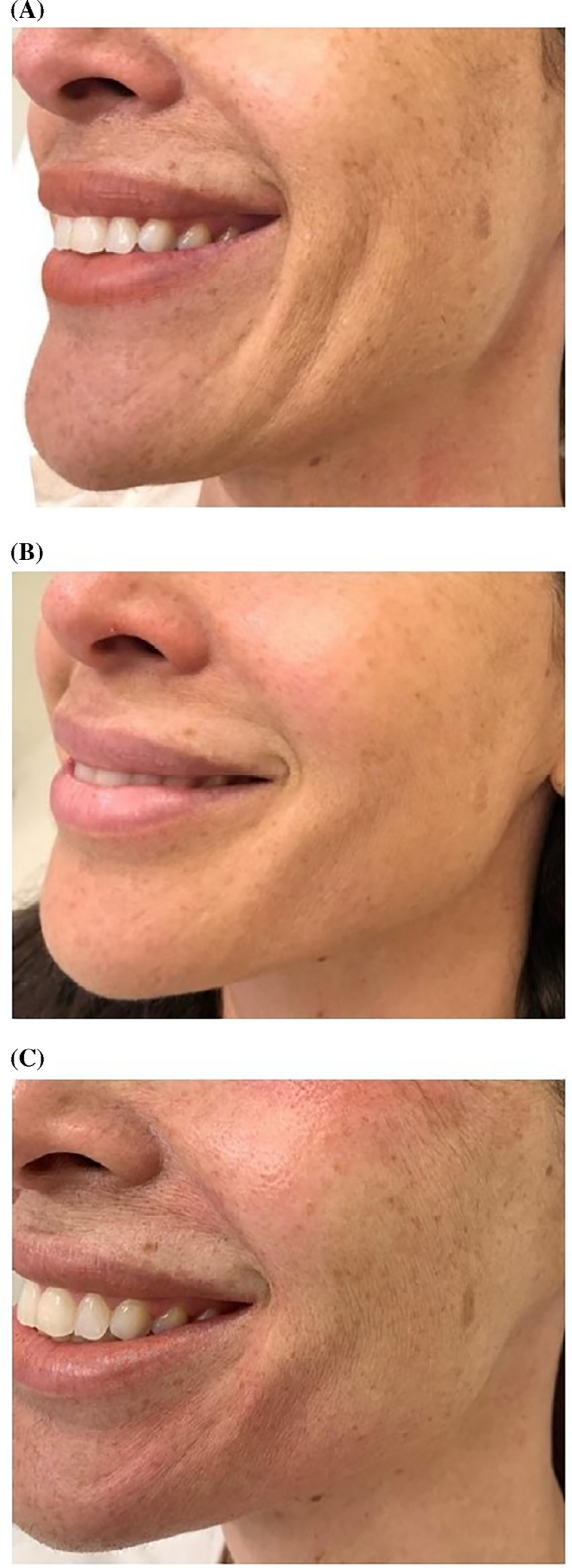
Forty‐three‐year‐old women treated with one syringe in each “lateral face” area. Pretreatment A; at 2 months after treatment B; at 8 months after treatment C

A total of 108 subjects were treated with Juvéderm Volite injectable gel from May 13, 2018 to October 11, 2019 at the Les Peaux Dermatological Clinic. The total number of treatment sessions consisted of 159, with a mean follow‐up time of 300.3 days. Figure 2 shows the results observed in a patient during an 8‐month follow‐up period.

Table 1 shows characteristics of the study subjects according to gender and age groups, stratified by needle use and cannula use. Patients were primarily female (91.7%), with a mean age of 53.3 years (SD = 11.8).

**TABLE 1:** Characteristics of patients stratified by injection technique (needle vs cannula) and age group

Patient characteristics	Total (n = 108)		Cannula use (n = 47)		Needle use (n = 79)		Up to 40 years (n = 13)		Over 40 years (n = 95)	
	n	%	n	%	N	%	N	%	n	%
Gender										
Male	9	8.3	2	4.3	7	8.9	3	23.1	6	6.3
Female	99	91.7	45	95.7	72	91.1	10	76.9	89	93.7
Age										
Mean (SD)	53.3	11.8	54.5	10.4	53.3	12.1	34.4	5.5	55.9	9.9
Under 40 years	13	12.0	4	8.5	9	11.4	13	100.0	‐	‐
41‐50 years	32	29.6	13	27.7	25	31.6	‐	‐	32	33.7
51‐60 years	26	24.1	13	27.7	17	21.5	‐	‐	26	27.4
61‐70 years	30	27.8	14	29.8	22	27.8	‐	‐	30	31.6
Over 70 years	7	6.5	3	6.4	6	7.6	‐	‐	7	7.4

Table 2 shows the characteristics of each treatment session with Juvéderm Volite. Of the total 159 sessions, injections with needles occurred in 116 (73.0%) and with cannulas (sizes 25G, 27G, and 30G) in 58 (36.5%). The concomitant use of a needle and cannula occurred in 15 sessions (9.4%); 66.7% of the patients in the study received one Volite syringe per treatment session. Of the patients treated with a needle, 57.8% received one syringe per session; the same amount of product was used in 75.9% of those treated with a cannula; 93.8% of patients who received one syringe per session were aged up to 40 years; 45.9% of the study subjects were treated in the perioral region, followed by the lateral face (28.9%), lips (13.8%), neck and infraorbital region (10.1% each), and crow's feet and décolletage (9.4% each).

**TABLE 2:** Treatment characteristics with Juvéderm Volite

Treatment characteristics	Total (n = 159)		Cannula (n = 58)		Needle (n = 116)		Up to 40 years (n = 16)		Over 40 years (n = 143)	
	N	%	N	%	n	%	n	%	n	%
Number of Volite syringes										
1	106	66.7	44	75.9	67	57.8	15	93.8	91	63.6
2	40	25.2	10	17.2	36	31.0	1	6.3	39	27.3
3	12	7.5	4	6.9	12	10.3	‐	‐	12	8.4
4	1	0.6	‐	‐	1	0.9	‐	‐	1	0.7
Treatment area										
Perioral region	73	45.9	26	44.8	50	43.1	1	6.3	72	50.3
Lateral face	46	28.9	‐	‐	46	39.7	‐	‐	46	32.2
Lips	22	13.8	16	27.6	6	5.2	1	6.3	21	14.7
Neck	16	10.1	‐	‐	16	13.8	6	37.5	10	7.0
Infraorbital	16	10.1	16	27.6	‐	‐	2	12.5	14	9.8
Décolletage	15	9.4	‐	‐	15	12.9	2	12.5	13	9.1
Crow's feet	15	9.4	2	3.4	14	12.1	1	6.3	14	9.8
Forehead	6	3.8	3	5.2	3	2.6	1	6.3	5	3.5
Hands	3	1.9	‐	‐	3	2.6	2	12.5	1	0.7
Glabella	2	1.3	‐	‐	2	1.7	‐	‐	2	1.4
Abdomen scar	1	0.6	‐	‐	1	0.9	‐	‐	1	0.7
Acne Scar	1	0.6	‐	‐	1	0.9	‐	‐	1	0.7
Cannula use	58	36.5	58	100.0	15	12.9	4	25.0	54	37.8
Needle use	116	73.0	15	25.9	116	100.0	12	75.0	104	72.7
Combined treatment										
Other hyaluronic acid filler	57	35.8	25	43.1	37	31.9	3	18.8	54	37.8
Botulinum toxin	26	16.4	12	20.7	18	15.5	3	18.8	23	16.1
Poly‐l‐lactic acid	13	8.2	7	12.1	6	5.2	1	6.3	12	8.4
Calcium hydroxyapatite	13	8.2	9	15.5	5	4.3	‐	‐	13	9.1

Table 3 shows the mean number of syringes used per area. Of the treatment areas shown in Figure 1A, the lateral face was the area that required the most amount of product (mean: 1.3; SD: 0.5) and the glabella, the lowest (mean: 0.4; SD: 0.1).

**TABLE 3:** Mean number of Volite syringes per treatment area

Treatment area	Total (n = 159)		Cannula use (n = 58)		Needle use (n = 116)		Up to 40 years (n = 16)		Over 40 years (n = 143)	
	N	%	n	%	n	%	n	%	n	%
Perioral region	73	45.9	26	44.8	50	43.1	1	6.3	72	50.3
Mean number of syringes (SD)	1.0	0.2	1.0	0.4	1.0	0.2	1.0	‐	1.0	0.2
Lateral face	46	28.9	‐	‐	46	39.7	‐	‐	46	32.2
Mean number of syringes (SD)	1.3	0.5	‐	‐	1.3	0.5	‐	‐	1.3	0.5
Lips	22	13.8	16	27.6	6	5.2	1	6.3	21	14.7
Mean number of syringes (SD)	0.9	0.2	0.9	0.2	0.9	0.2	1.0	‐	0.9	0.2
Neck	16	10.1	‐	‐	16	13.8	6	37.5	10	7.0
Mean number of syringes (SD)	1.0	0.1	‐	‐	1.0	0.1	1.0	0.0	1.0	0.2
Infraorbital region	16	10.1	16	27.6	‐	‐	2	12.5	14	9.8
Mean number of syringes (SD)	0.9	0.2	0.9	0.2	‐	‐	1.0	0.0	0.9	0.2
Décolletage	15	9.4	‐	‐	15	12.9	2	12.5	13	9.1
Mean number of syringes (SD)	1.0	0.3	‐	‐	1.0	0.3	1.5	0.7	1.0	0.1
Crow's feet	15	9.4	2	3.4	14	12.1	1	6.3	14	9.8
Mean number of syringes (SD)	1.0	0.1	0.8	0.4	0.9	0.2	1.0	‐	1.0	0.1
Forehead	6	3.8	3	5.2	3	2.6	1	6.3	5	3.5
Mean number of syringes (SD)	1.0	0.5	1.2	0.8	0.9	0.2	1.0	‐	1.0	0.6
Hands	3	1.9	‐	‐	3	2.6	2	12.5	1	0.7
Mean number of syringes (SD)	1.0	0.0	‐	‐	1.0	0.0	1.0	0.0	1.0	‐
Glabella	2	1.3	‐	‐	2	1.7	‐	‐	2	1.4
Mean number of syringes (SD)	0.4	0.1	‐	‐	0.4	0.1	‐	‐	0.4	0.1
Abdomen surgical scar	1	0.6	‐	‐	1	0.9	‐	‐	1	0.7
Mean number of syringes (SD)	2.0	‐	‐	‐	2.0	‐	‐	‐	2.0	‐
Acne scar	1	0.6	‐	‐	1	0.9	‐	‐	1	0.7
Mean number of syringes (SD)	1.0	‐	‐	‐	1.0	‐	‐	‐	1.0	‐

With regard to combining treatments in the same Volite treatment session, the use of another hyaluronic acid dermal filler was the most frequently observed (35.8%), followed by botulinum toxin (16.4%), poly‐l‐lactic acid (8.2%), and calcium hydroxyapatite (8.2%) (see Table 2).

The need for subsequent treatment is presented in Tables 4 and 5, stratified by patient characteristics and areas of treatment, respectively, according to the injector's clinical observation.

**TABLE 4:** Need for subsequent treatment stratified by age group

	N	%	Mean time (months)
Total sample^a^			
Initial treatment	177	100.0	‐
Touch‐up treatment	15	8.5	1.8
Repeat treatment	16	9.0	5.5
Men			
Initial treatment	10	100.0	‐
Second treatment	‐	‐	‐
Repeat treatment	‐	‐	‐
Women			
Initial treatment	167	100.0	‐
Touch‐up treatment	15	9.0	1.8
Repeat treatment	16	9.6	5.5
Up to 40 years			
Initial treatment	15	100.0	‐
Touch‐up treatment	‐	‐	‐
Repeat treatment	1	6.7	6.9
Over 40 years			
Initial treatment	162	100.0	‐
Touch‐up treatment	15	9.3	1.8
Repeat treatment	15	9.3	5.4

^a^ Eight patients received a second treatment after touch‐up or repeat treatment.

**TABLE 5:** Need for subsequent treatment stratified by treatment areas

	n	%	Mean time (months)
Perioral region			
Initial treatment	53	100.0	‐
Touch‐up treatment	8	15.1	1.9
Repeat treatment	6	11.3	4.7
Lateral face			
Initial treatment	34	100.0	‐
Touch‐up treatment	4	11.8	1.5
Repeat treatment	6	17.6	6.5
Lips			
Initial treatment	22	100.0	‐
Touch‐up treatment	‐	‐	‐
Repeat treatment	‐	‐	‐
Neck			
Initial treatment	14	100.0	‐
Touch‐up treatment	‐	‐	‐
Repeat treatment	2	14.3	6.7
Infraorbital region			
Initial treatment	15	100.0	‐
Touch‐up treatment	1	6.7	2.6
Repeat treatment	‐	‐	‐
Décolletage			
Initial treatment	14	100.0	‐
Touch‐up treatment	‐	‐	‐
Repeat treatment	1	7.1	3.9
Crow's feet			
Initial treatment	13	100.0	‐
Touch‐up treatment	2	15.4	1.8
Repeat treatment	‐	‐	‐
Forehead			
Initial treatment	5	100.0	‐
Touch‐up treatment	‐	‐	‐
Repeat treatment	1	20.0	3.9
Hands			
Initial treatment	3	100.0	‐
Touch‐up treatment	‐	‐	‐
Repeat treatment	‐	‐	‐
Glabella			
Initial treatment	2	100.0	‐
Touch‐up treatment	‐	‐	‐
Repeat treatment	‐	‐	‐
Abdomen surgical scar			
Initial treatment	1	100.0	‐
Touch‐up treatment	‐	‐	‐
Repeat treatment	‐	‐	‐
Acne scar			
Initial treatment	1	100.0	‐
Touch‐up treatment	‐	‐	‐
Repeat treatment	‐	‐	‐

Of the 108 subjects, 8.5% received touch‐up treatment: Of those older than 40 years of age, the rate was 9.3%; of the women who participated in the study, 9.0% received touch‐up. Among male subjects and those aged up to 40 years, there was no need for touch‐up. With regard to the area of treatment, touch‐up injections were most commonly observed in the crow's feet area (15.4%), followed by the perioral region (15.1%). The mean time of touch‐up ranged from 1.5 months for the treatment of the lateral face and 2.6 months for the infraorbital region.

Repeat treatment was performed in 9.0% of the subjects in the sample. Of those older than 40 years of age, the rate was 9.3% and 6.7% in those younger than 40. Of the women who participated in the study, 9.6% received repeat treatment. With regard to areas that required repeat treatment, the forehead was the most commonly observed area (20.0%), followed by the lateral face (17.6%). Repeat treatment was performed with mean intervals that ranged from 3.9 months after initial treatment in the décolletage area and 6.7 months in the forehead.

The safety of Juvéderm Volite was assessed over a mean follow‐up period of 300.3 days. There were no cases of visible lumps, nodule formation, or late hypersensitivity. In order to generate a hypothesis to explain the different patterns of use of Juvéderm Volite, the study analyzed the associations between different variables such as the number of Volite syringes used and injection technique using a needle vs a cannula. The results are presented in Tables 6 and 7, respectively.

**TABLE 6:** Hypothesis tests to verify correlation between treatment characteristics and number of Volite syringes

	Number of syringes		*P*‐value
	Mean	SD	
Gender			
Male	1.0	0.0	**.019**
Female	1.4	0.6	
Age			
Up to 40 years	1.0	0.1	**.012**
Over 40 years	1.4	0.6	
Cannula use			
Yes	1.3	0.5	.320
No	1.4	0.7	
Needle use			
Yes	1.5	0.6	**<.001**
No	1.0	0.2	
Perioral region			
Yes	1.7	0.7	**<.001**
No	1.1	0.3	
Lateral face			
Yes	2.0	0.6	**<.001**
No	1.1	0.2	
Combined treatment with other HA filler			
Yes	1.3	0.4	.559
No	1.4	0.7	
Combined treatment with botulinum toxin			
Yes	1.3	0.4	0.990
No	1.4	0.6	

*Note*: The analysis only considered cases in which treatment areas and combined treatments occurred in at least 15% of the sessions.

The numbers in bold had Statistical significance.

**TABLE 7:** Hypothesis tests to verify characteristics associated with the material used in applications

	Use of cannula				*P*‐value	Use of needle				*P*‐value
	No		Yes			No		Yes		
	N	%	n	%		n	%	n	%	
Gender										
Male	7	77.8	2	22.2	.294	2	22.2	7	77.8	1.000
Female	54	54.5	45	45.5		27	27.3	72	72.7	
Age										
Up to 40 years	9	69.2	4	30.8	.490	4	30.8	9	69.2	.744
Over 40 years	52	54.7	43	45.3		25	26.3	70	73.7	
Perioral region as the treatment area										
Yes	30	56.6	23	43.4	1.000	11	20.8	42	79.2	.236
No	31	56.4	24	43.6		18	32.7	37	67.3	
Lateral face as the treatment area										
Yes	22	64.7	12	35.3	.337	0	0.0	34	100.0	**<.001**
No	39	52.7	35	47.3		29	39.2	45	60.8	
Associated treatment with other hyaluronic acid filler										
Yes	22	48.9	23	51.1	.251	14	31.1	31	68.9	.533
No	39	61.9	24	38.1		15	23.8	48	76.2	
Associated treatment with botulinum toxin										
Yes	12	52.2	11	47.8	.816	5	21.7	18	78.3	.720
No	49	57.6	36	42.4		24	28.2	61	71.8	

*Note*: The analysis only considered cases in which treatment areas and combined treatments occurred in at least 15% of the sessions.

The numbers in bold had Statistical significance.

There was a significant increase in the number of Volite syringes used to treat female patients (*P* = .019), aged over 40 years (*P* = .012), in the perioral region (*P* < .001) and lateral face (*P* < .001; Table 6), with the use of a needle (*P* < .001). There was a significant correlation between needle use and treatment of the lateral face (all patients who underwent treatment of the lateral face were injected with a needle at least once; *P* < .001). The rate among those receiving treatment in other areas was 62.2%. No significant correlations were observed with the use of cannula (Table 7).

## 4 DISCUSSION

The aim of this study was to investigate the use of Juvéderm Volite to improve skin quality in a clinical setting in Brazil. This new *hyaluronic acid*‐based *dermal filler, currently available* in the global non‐invasive aesthetic treatment market, uses the relatively new *Vycross* Technology, designed to provide optimal results with a lower incidence of treatment‐related adverse events. However, to our best knowledge, there have been no studies published to date describing the practical use of this specific hyaluronic acid with a cannula. Thus, this study adds important knowledge to clinical practice.

The primary outcome measure of this study was the need for subsequent treatment in Juvéderm Volite cosmetic procedures. Two variables were defined: touch‐up treatment (≤3 months) and repeat treatment (≥3 months). Follow‐up visits that occurred within 3 months after initial treatment were classified as touch‐up treatment and those occurring after this period were classified as repeat treatment. The 3‐month time limit was based on the clinical experience of the author. Previous studies evaluating the efficacy of Juvéderm Volite for improving skin quality attributes have described touch‐up treatment 30 days after initial treatment,^8^ at day 45,^10^ and repeat treatment 9 months after last treatment (initial or touch‐up).^8^

The retrospective design of the present study did not permit the objective assessment of the efficacy of Juvéderm Volite to improve overall skin quality attributes, such as roughness, fine lines, hydration, and elasticity. The efficacy of Juvéderm Volite, however, has been previously reported in the literature in at least two studies.^8^, ^9^, ^10^

Cavallini et al (2019) conducted a prospective, nonrandomized, open‐label study to assess the effect of facial treatment with Juvéderm Volite on skin texture. Women presenting fine to moderate facial lines, skin with low hydration and brightness, and signs of chronological aging and photoaging received initial treatment and touch‐up treatment at day 45 if required. The need for touch‐up was observed in 20.0% of the subjects included in the sample. Mean improvements in skin texture were 25.9% ± 9.2% at day 45 and 30.7% ± 18.2% at month 6, measured using an optical scanning device. Response rates were higher in cheeks (27.6% at day 45 and 33.3% at month 6), compared to neck (23.6% at day 45 21.6% at month 6) and perioral region (17.8% at day 45 and 24.0% at month 6).^10^

Niforos et al (2019) conducted a prospective, single‐arm study to evaluate the safety and effectiveness of Juvéderm Volite to improve skin quality attributes such as surface smoothness and hydration. Subjects with moderate to severe cheek skin roughness received initial treatment, touch‐up treatment 30 days later and optional repeat treatment 9 months after last treatment (initial or touch‐up). Of 131 subjects treated, 31 (23.7%) received touch‐up treatment at days 30 and 62 (47.3%) received repeat treatment at month 9. The response rate assessed with the validated 5‐point photonumeric Allergan Skin Roughness Scale was 96.2% at month 1, 76.3% at month 4, 34.9% at month 6, 15.8% at month 9, and 87.1% after repeat treatment.^8^

In the present study, clinical observation was used to understand the role of Juvéderm Volite on the improvement of skin quality. Four patients did not return to clinical assessment; however, they were not excluded from the analysis to enable assessment of other characteristics related to product use. Of the 108 subjects who received initial treatment, only 8.5% received touch‐up and 9.0% received repeat treatment following injectors' choice, during the entire period of the study. Need for repeat treatment was most frequently observed in the crow's feet area (15.4%), followed by the perioral region (15.1%). The fact that crow's feet required a greater number of repeat treatments than other areas may be justified by the fact that they are hyperkinetic facial lines. The greater need for repeat treatment also observed in the perioral region (15.1%) is consistent with what was previously reported by Cavallini et al (2019), reinforcing the understanding that the treatment response for the perioral region may be less effective.^10^

The significantly lower number of touch‐up and repeat treatments described by the investigator in the present study, compared to others published in the literature, may be justified by the following facts: As prior cosmetic procedures were not considered as exclusion criteria for participation, patients who had been treated with dermal fillers or undergone other skin rejuvenating procedures over the past 6 months *were included in the cohort*. Also, 35.8% of patients received at least one combined treatment in the same session.

The safety of Juvéderm Volite was assessed during the mean follow‐up period of 300.3 days. There were no reports of visible or palpable lumps, nodules, or late hypersensitivity. Transient adverse events commonly observed in the subsequent days after a procedure, such as edema, bruising, and hematoma, were not reported due to the lack of available data.

In his study, Niforos et al (2019) reported the occurrence of treatment‐related adverse events in 15.3% of patients. Events reported were injection site mass (9.2%), injection site bleeding (3.1%), injection site hematoma (2.3%), injection site erythema (0.8%), and injection site nodule (0.8%).^8^

Ogilvie et al (2020) published an expert consensus recommending Volite space injections approximately 0.5 to 1.0 cm apart, with volumes ranging from 0.01 to 0.05 mL per injection. Small adjustments can be made to the volume and spacing according to specific skin regions and patient characteristics. The present author agrees with the spacing of injections recommended in the study for most treatments. Nevertheless, in procedures aimed at improving rhytids and linear depressions (glabella lines, vertical forehead lines, and crow's feet and horizontal neck lines), space injections should be closer ranging from 0.2 to 0.3 cm apart. Although the expert consensus recommends injection volumes between 0.01 and 0.05 mL at each site, the author's experience suggests that in treatments carried out with the use of a needle the volume injected should not exceed 0.01 mL, as it would be almost impossible to inject 0.05 mL of product into the deep dermis without producing a visible lump or nodule at the site of injection.^11^

Different treatment patterns using Juvéderm Volite were also revealed in the study. The association between different patient characteristics and individualized treatment plans was assessed by the investigator, and some statistically significant differences were observed: female patients older than 40 years of age who received treatment of the perioral region and lateral face, with the use of a needle in at least one session, required a greater number of Volite syringes. The use of a needle was associated with the treatment of the lateral face. This may be justified by the fact that this is a larger area of treatment requiring a greater number of injections. It is also worth noting that subjects who underwent treatment of the lateral areas of the face frequently combined other treatments in the same Volite session. In line with previous studies, treatment of this area was carried out with a needle in all cases. As the skin is thicker in this region, it is technically easier to inject the product into the deep dermis with a needle than a cannula. However, in areas where skin is thinner such as in the infraorbital region, lateral forehead depressions, and perioral region with severe photodamage, it is recommended to place the filler in the superficial subcutaneous plane with a cannula to ensure greater comfort and safety for patients while at the same time reducing the risk of complications such as visible lumps and nodule formation. Although treatment of the neck with Volite has demonstrated to be one of the areas with the most satisfying results, it is recommended to treat only the horizontal neck lines and not the entire area, as this can lead to visible or palpable *unintended* mass. In the opinion of the author, cases in which patients require full treatment of the neck area it is recommended to use collagen biostimulators such as poly‐l‐lactic acid or calcium hydroxyapatite.

At the Les Peaux Dermatological Clinic, the use of a needle was favored over a cannula for treatments with Volite; however, the latter may be used to treat the forehead, infraorbital, perioral, and mouth regions. Statistical analysis reinforces these patterns, showing a significant association between the use of a needle and specific areas of treatment. No other study supporting such correlations has been found to date. This relationship, therefore, requires further investigation. The study did not find significant differences with regard to the two injection techniques in terms of efficacy and safety of Juvéderm Volite. Nevertheless, injections performed with a cannula apparently require a greater amount of product to treat the same area compared with a needle. The higher correlation between the use of a cannula and less amount of product (one syringe per session) described in the study may be explained by the fact that cannulas are usually preferred over needles to treat smaller areas as, for example, the perioral and infraorbital regions. With that being said, as Juvéderm Volite is a hyaluronic acid‐based product intended for intradermal injection, the use of a needle demonstrated to be more suitable. As mentioned before, this is not always possible in cases where skin is characterized by severe photoaging and deep furrowing.

Although the study makes a valuable contribution to the existing body of knowledge, there are a few limitations that must be acknowledged. Major limitations were related to the need for touch‐up treatment and/or repeat treatment, as well as the use of needle or cannula, which were solely based on the injectors' choice. These definitions provide results based on subjective assessments and limits comparison with the results from other studies. However, the results reported in the present study may be useful to serve as a basis for further investigations. In addition, it is important to highlight that the injector has extensive experience with the product line using Vycross technology (Allergan Inc.), having used over 15 000 syringes (Voluma, Volift, and Volbella) in a broad range of treatments over the years, before the launch of Volite in the Brazilian market.

A second limitation is associated with the retrospective design of the study. *As participants were not randomly selected*, *the sample frame* may not be representative of the population of interest. Collecting data from medical records retrospectively will depend largely on the timeliness and accuracy of the attending physician, which in turn may be considered less reliable.

In summary, this study has shown that Juvéderm Volite is a useful tool to improve skin quality and, according to clinical observation, requires fewer and less frequent maintenance treatments. The product can be injected using both cannula and needle. Besides the ability to improve skin quality, the major contribution of the present analysis was to attest the safety profile of the new cross‐linked HA gel, in which no adverse events were observed during the entire period of the study.

## CONFLICT OF INTEREST

Daniel Dal'Asta Coimbra is a speaker from Allergan (now AbbVie); however, no funding was provided for the conduction of this study.

## AUTHOR CONTRIBUTIONS

Conceptualization: Daniel Dal'Asta Coimbra

Writing—original draft preparation: Daniel Dal'Asta Coimbra

Writing—review and editing: Daniel Dal'Asta Coimbra

## TRANSPARENCY STATEMENT

Daniel Dal'Asta Coimbra affirms that this manuscript is an honest, accurate, and transparent account of the study being reported, that no important aspects of the study have been omitted, and that any discrepancies from the study as planned (and, if relevant, registered) have been explained.

## Data Availability

The authors confirm that the data supporting the findings of this study are available within the article or its supplementary materials.
